# Validity and reliability of a finger training tool for assessing metacarpal phalangeal joint ranges of motion in asymptomatic participants

**DOI:** 10.1038/s41598-024-71094-y

**Published:** 2024-08-29

**Authors:** P. Kuvijitsuwan, J. Klaphajone, P. Singjai, T. Kumpika, N. Thawinchai, C. Angkurawaranon, C. Aramrat, K. Utarachon

**Affiliations:** 1 Department of Physical Rehabilitation Medicine, Faculty of Medicine, Chiang Mai University, Chiang Mai, Thailand; 2 Department of Physics and Materials Science, Faculty of Science, Chiang Mai University, Chiang Mai, Thailand; 3 Department of Physical Therapy, Faculty of Associated Medical Sciences, Chiang Mai University, Chiang Mai, Thailand; 4 Department of Family Medicine, Faculty Medicine, Chiang Mai University, Chiang Mai, Thailand

**Keywords:** Manual goniometer, Range of motion, Therapeutic device, Minimal detectable difference, Therapeutics, Mechanical engineering, Health care, Health occupations, Engineering

## Abstract

This pilot study aims to evaluate concurrent validity using the goniometer as a reference tool and test–retest reliability of flexion of metacarpal phalangeal joint (MCP) measurements taken from a finger training device (air-guitar system) in healthy participants. There were ten self -reported asymptomatic participants recruited to test the devices. The measurements of all metacarpophangeal joints of the dominant hands were conducted using a finger goniometer and the air-guitar system. Two measuring sessions were conducted on the same day. The concurrent validity of the air-guitar indicated by strong concordance correlation coefficient (0.62–0.90) with the goniometer and mean difference (approximately 1°) between the two instruments are well below the limit of 5°. The test–retest reliability of MCP measurements from the air-guitar glove (0.82–0.99) was acceptable as a clinically meaningful measurement tool as the intraclass correlation coefficients were higher than 0.7. The standard error of measurement and minimal detectable change of the air-guitar are similar to those of the goniometer. The air-guitar tracking features, when used as a home-based therapy tool, may assist in monitoring change of MCP flexion over a time course with good reliability and strongly associated with the measurements from the goniometer.

## Introduction

Hand impairments can result from aging, injuries, and several diseases such as musculoskeletal disorders, neurological disorders, and developmental disabilities. The prevalence of hand disabilities was reported at 13.6% (7.2% in men and 17.8% in women) in people aged over 55 years^1^. With the increase in use of cellphones and computers, the incidence of hand disorders due to musculoskeletal conditions also increased, from 206.5 cases per 100,000 individuals per year in 2001 to 222.5 cases in 2011^2^. Additionally, there are now more people suffering from neuromuscular problems. The overall incidence rate of stroke from 1998 to 2017 was 109.21 per 100,000 individuals per year^3^. Approximately 50–80% of these suffered from upper limb impairments including hands^4^. People with difficulty in grips and grasps are more likely to have limited functions for activities of daily living (ADL), hence independent living. There are several approaches, such as orthoses and occupational therapy, for regaining ADL functions such as grips and grasps based on neuroplasticity concept. Individual needs and conditions are considered to personalize an individual’s rehabilitation program in the hospital and at home to optimize outcomes. The continuation of therapy is crucial for functional recovery. In particular, the first 6 months after stroke onset are regarded as the ‘golden period’ due to spontaneous recovery^5,6^. However, many patients had trouble getting the right therapeutic and orthotic guidance, which made injuries more likely^7^.Also, in the absence of motivation, many patients were unable to continue practicing consistently and intensively. Moreover, travel expenses for several hospital visits could be a financial burden for the patients and caregivers. Another factor is the workload of the therapists resulting in long waiting time for the patients. As a result, their functional outcomes are more likely to fall short of their initial potential. An effective home-based therapeutic program has become a potential to overcome some of these barriers.

Regarding home-based therapeutic program, patients adhere to written instructions for the therapist’s recommended exercises and use everyday objects, such as putty, for strength training. This approach is cost-effective provided that certain levels of intensity, repetition, and concentration are achieved to maximize the possibility of hand movement recovery^8–11^. However, many patients complained of receiving inadequate assistance, which led to a slower rate of functional improvement than anticipated^7^. This affects motivation of the patients, intensity and repetition of therapeutic exercises, hence functional outcomes.

Researchers all over the world are currently developing robot-assisted treatment and tools for assisting in functional rehabilitation using either an active exoskeleton or demanding, serious therapeutic games. Commercially available stationary hand rehabilitation devices include HandTutor^12^, EsoGlove Pro^13^, Gloreha Sinfonia^14^, Hand of Hope^15^ and Syrebo Hand^16^ etc. These are only accessible in clinical settings because of their high cost and ongoing maintenance requirements such as system calibration, pneumatic or hydraulic actuators. To our knowledge, the only approved home-based therapeutic device is MusicGlove^17^. It is merely intended to facilitate and motivate grips through an interactive music game with low maintenance. There are no features for monitoring development toward the goals of rehabilitation such as functional range of motion, or muscle strengthening which affect ADL^18^. These features can assist with telerehabilitation by improving long-distance therapy services for those who have travel difficulties, reducing long-term travel expenditures and the workload for health care professionals. At the same time, users can be motivated through continuous feedback from the device. Also, the survey carried out in 2014 urged the need for home-based devices for therapy and ADL support to be more cost-effective and efficient^19^. Therefore, Air-guitar system was designed to integrate a measuring device, a serious game, and a USB controller at low cost and low maintenance to support hand therapy at home and promote long-term continuity.

### Device design: air-guitar system

**Fig. 1:**
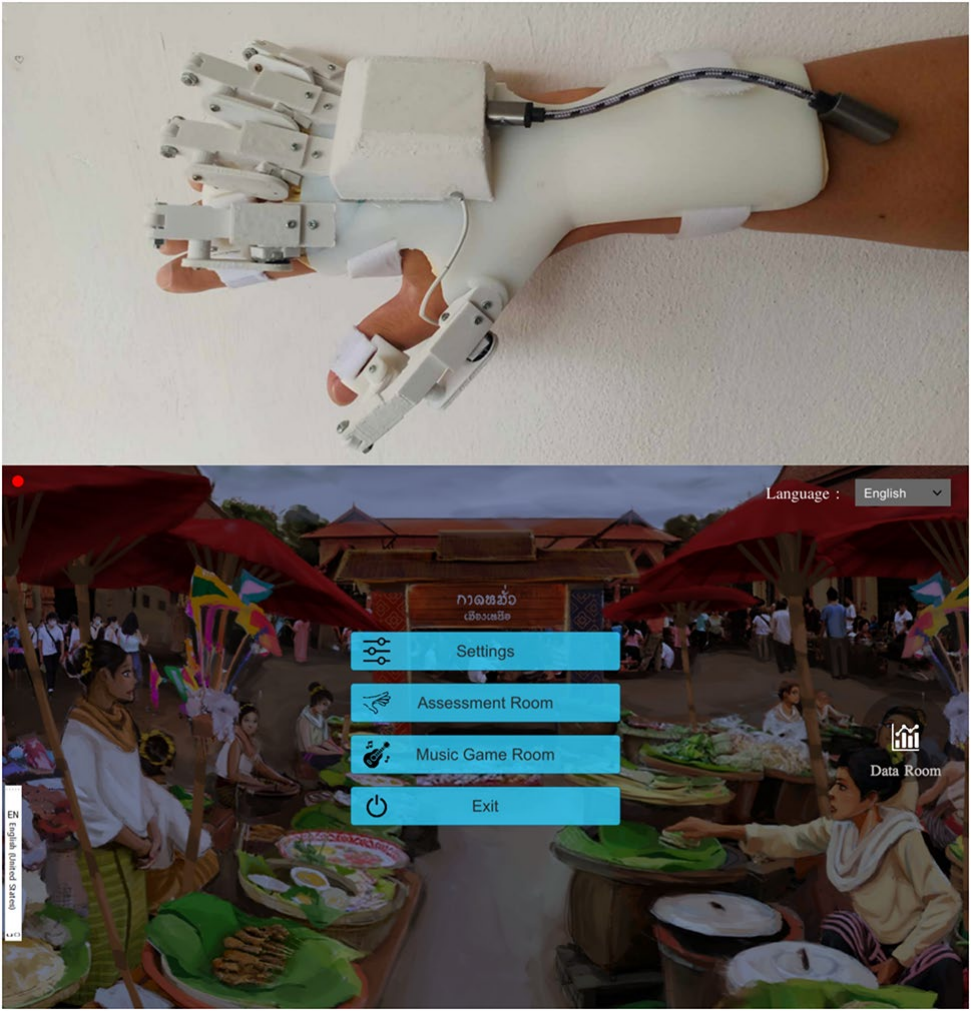
A new hand therapeutic air-guitar device (Patent submission on 2303001869 July 10th, 2023).

The air-guitar system focuses on finger range of motion exercises with adjustable resistance to facilitate hand rehabilitation (Fig. 1). The system comprises a joint-monitoring glove with adjustable resistance for muscle strengthening, a USB-controller, a serious game with grip exercises and instruction available in English and Thai, and specifically designed therapeutic music for motivation. The glove monitors the movement of metacarpophalangeal joints (MCP), which are crucial for grip and grasp functions^20^. The software features an assessment room and a therapy game room (Fig. 1). In the assessment room, the air-guitar hand device records the static active range of motion, much like a finger goniometer does in clinical practice. The therapy room provides a music game featuring motivational music therapy where the user can practice gripping in response to computer-generated visual cues on the screen. The system would provide a real-time score for timely and correctly completed movements it recognized. A score and MCP range of motion for each finger are reported at the end of the training session to support specific treatment plans and track progress. Therefore, it is important to evaluate the concurrent validity and the test–retest reliability of the system. The standard goniometer technique in a clinical setting is regarded as reference and comparison with previous literature.

This preliminary study aims to evaluate the test–retest reliability of maximum flexion of MCP measurements from a hand therapeutic device (air-guitar system) in healthy participants, as well as the concurrent validity of the device using the goniometer as a reference.

## Materials and methods

### Participants

According to Bonnett^21^, sample size (n) for ICC is calculated from minimum acceptable reliability ICC = 0.7, expected reliability ICC = 0.95, significant level = 0.05, power = 80%, number of repetitions per subject (k) = 2 and expected dropout rate 0%, then n = 10.Ten healthy right-handed participants were recruited at Chiang Mai University, Thailand. They were aged between 18 and 60 years without medical history or injuries to the upper limbs or neuromuscular systems based on self-report. The same clinician with ten years of experience conducted the measurements using the air-guitar and the finger goniometer in two sessions. The Chiang Mai University Faculty of Medicine’s Research Ethics Committee gave its approval to the study protocol (REH-2565-08821). A Certificate of Ethical Approval (number 356/2022) was issued on October 17, 2022 (Supplementary information). All participants gave their written informed consent after being informed of the study's aims and procedures. The study adhered strictly to the standard and guidelines including principles of the declaration of Helsinki.

### Equipment

#### Goniometer

**Fig. 2:**
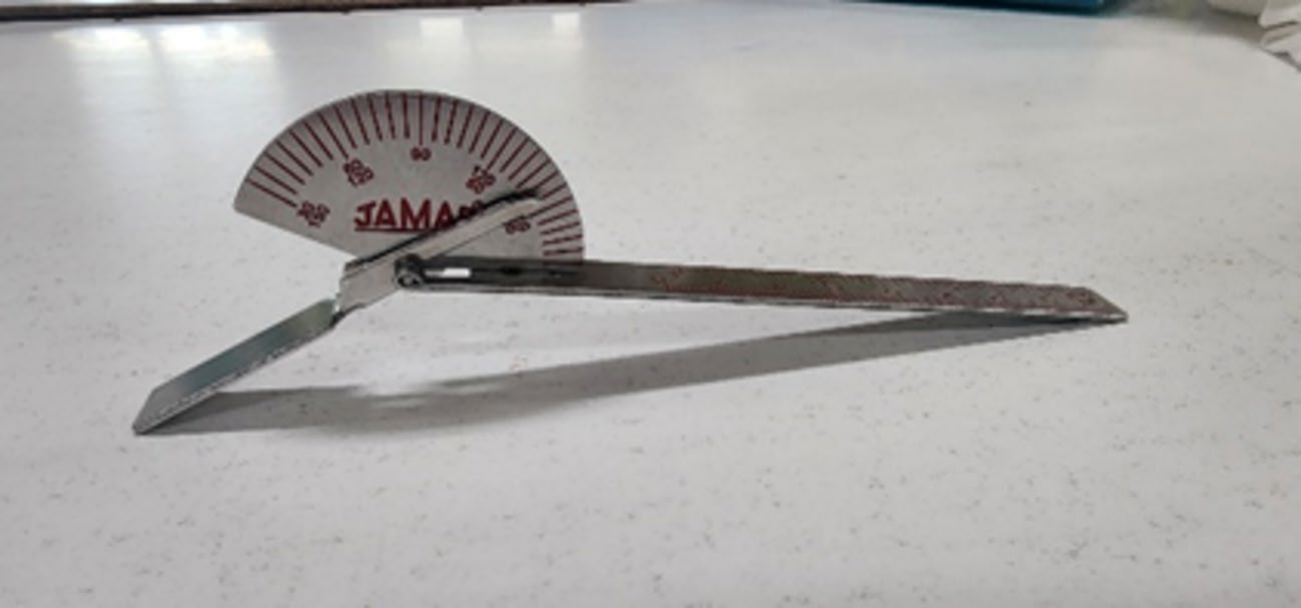
A stainless-steel Jamar finger goniometer.

A stainless-steel Jamar finger goniometer (JAMAR®, Patterson Medical Supply, CA) equipped with both long and short arms, allows for precise measurement of finger movement with an accuracy of up to five degrees. Measurements ranged from 120° of flexion to 30° of hyperextension (Fig. 2).

#### Air-guitar system

The “Air-guitar system” is a home-based finger training apparatus comprising a plastic wrist-hand orthosis with individual finger extensions and integrated software for serious gaming and measurement of MCP joints (Fig. 1). Developed at Chiang Mai University in Chiang Mai, Thailand, this system is currently in the pre-clinical stage of development. The device has successfully met electrical safety standards for medical devices and has undergone rigorous usability and medical software testing.

**Fig. 3:**
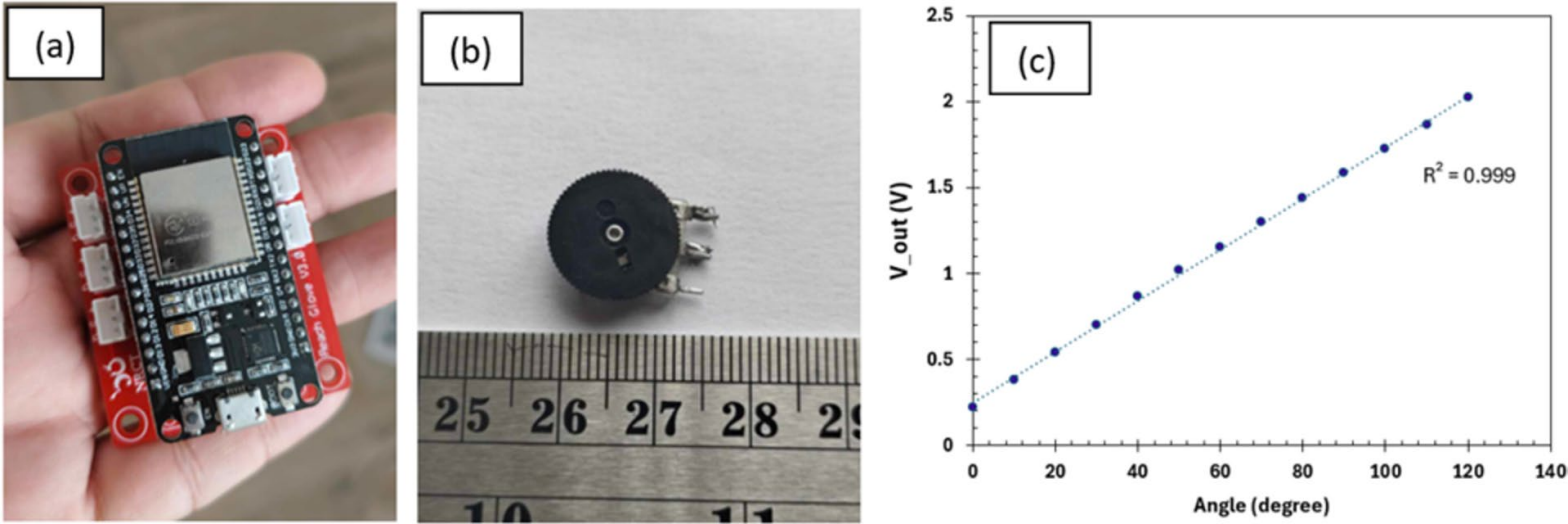
(**a**) ESP 32 on print circuit (**b**) potentiometer (**c**) relations of output voltage with angle.

The electronic circuit in the monitoring system is designed to connect the ESP32 microcontroller to the potentiometer 50 k plate sensors, as shown in Fig. 3a and b, respectively. The potentiometer functions as a variable resistor, changing resistance in accordance with the wiper position. Five potentiometers were used to place the angle sensors on each finger’s MCP. When a voltage (V_in) is applied, the position of the wiper affects the output voltage (V_out), which is proportionate to its location along the resistive track. Thus, the voltages determined the angles, as seen by the relationship between the angle and V_out in Fig. 3c. The air-guitar system is capable of recording angles between 0 and 135 degrees due to its design.

**Fig. 4:**
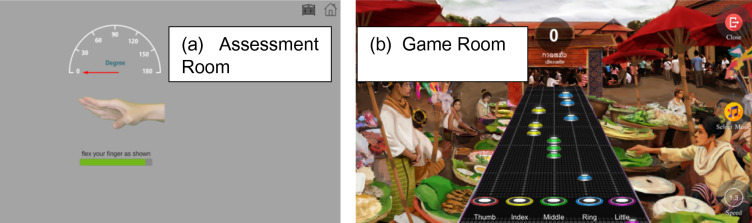
(**a**) Assessment room and (**b**) game room.

The plastic wrist-hand orthosis is available in three sizes—small, medium, and large—based on palm width. After selecting the appropriate size, participants wear the air-guitar device, as shown in Fig. 4. Soft Velcro straps secure the plastic structures to each finger, enabling independent finger movement. During the initial calibration process, the participant places his hand and fingers flat on a table to establish a neutral alignment of zero. Each user only needs to perform this calibration once, which facilitates ongoing patient monitoring at home.

The air-guitar device features two primary modes: the assessment mode (Fig. 4a), which measures the flexion range of the MCP joints, and the game mode (Fig. 4b), which promotes intensive finger-grip exercises through serious music games. Both modes record finger motion data for continuous comparison throughout rehabilitation. This study used only the assessment mode to evaluate the MCP joints’ active maximal flexion.

#### Protocol

After informed consent was obtained, the participant was brought to the motion capture room at the physiotherapy department at Chiang Mai University. A computer-generated order randomly assigned the participant to either the air-guitar or the goniometer group. After finishing the first session, the participant had an approximately 15-min break before starting the second session.

A clinician with 10 years of clinical experience performed the goniometric measurements, while a technician supported the use of the air-guitar system throughout the sessions. To ensure that the clinician and technician were blinded to each other’s readings, the clinician and the technician performed measurements independently. The technician fitted the air-guitar device on the patient, ensuring proper connectivity and adherence to the user manual. The technician assisted the participant in following the on-screen instructions, verified the device’s fit, recorded the measurements displayed on the screen, and documented them on the appropriate form. Additionally, the technician addressed any concerns regarding the air-guitar system, including any discomfort experienced by the participant.

### MCP joint measurements

For the goniometer group, the participant had his forearm in a neutral position and had his elbows on the table. The recommendation of the American Society for hand therapists for the finger joints was followed^22^. The clinician positioned the goniometer on the dorsal aspect and examined five MCP joints. The participant was instructed to flex their MCP joint separately, one at a time, and then the clinician read the measurement on the goniometer with 5° increment. If the goniometer indicated a space between the increments, 2.5° was read. After the measurement was taken, the participant was instructed to fully extend his MCP joint before fully flexing for next measurement. These steps were repeated for three measurements before proceeding to assess the other joints.

Using the air-guitar device, the active range of MCP joint flexion was also determined. The participant wearing the air-guitar device received assistance from the technician. The participant was given instructions to do the measurements as shown on the computer screen in the “Assessment room” mode. The technician recorded the readings on the screen.

All measurements were performed three times, consistent with clinical routine, by the same examiners. For further analysis, the mean value of these measurements was used. All measuring procedures were documented on a blank case record form to minimize the influence of previous readings. Measurements were repeated in the afternoon under identical conditions to evaluate the concurrent validity and test–retest reliability of the methods.

#### Data processing and analysis

All analyses were conducted using STATA/MP17 (StataCorp, 4905 Lakeway Drive, College Station, TX 77,845, USA). Descriptive data: age, weight, height, Body Mass Index (BMI) and angles are reported as mean and standard deviation (SD) with minimal and maximal values.

### Concurrent validity

The concurrent validity indicates the degree to which scores on the measure agree with scores on another measure taken at the same time. In this context, Lin’s concordance correlation coefficients (CCC) are calculated as CCC is the concordance between two readings: a new test or measurement and a gold standard test or measurement. This statistic evaluates the agreement between two methods measuring the same variable by measuring the variance from the 45° line across the origin (the concordance line)^23^. It can be used in small sample sizes of as few as ten. Bland and Altman plot is also presented for visualization with Mean difference and Limit of agreement between two methods. This information is helpful for clinical application.

In this study, the concurrent validity was determined between the goniometer and the air-guitar device for maximum flexion position of the MCP joint using CCC and the Bland Altman plot. The acceptable mean difference between two-method is set at 5° as this is an approximate variability of the standard goniometer readings of the joint angle measurements in the hand and the goniometer used can read precisely with 5°. This mean difference is also comparable to previous report^24^. The interpretation of CCC is still controversial. Akoglu suggested that CCC should be interpreted closely compared to other correlation coefficients, like Pearson’s correlation coefficient: 1 indicates perfect agreement, 0.8–0.9 indicates very strong agreement, 0.6–0.7 indicates moderate, 0.3–0.5 indicates fair, 0.1–0.2 indicates poor and 0 indicates non agreement^25,26^.

Mean difference and 95% limit of agreement were also calculated for Bland and Altman plot to visualize data^27^. All data points (6 pairs of data) from each participant were plotted to maximize the number of pairs of data to visualize any possible relationship between mean differences and the best possible true value. The differences between the goniometer and the air-guitar measurements were plotted against the mean of the two measurements to investigate any possible relationship between measurement error and the true value. The mean of the two measurements is the best estimate for the true value^28^. The use of a standard or reference method instead of the mean of the two measurements is controversial because a relation between difference and magnitude is likely, though there is none^29^. The regression lines and confidence interval limits were also presented to visualize the trend of proportional difference^30^.

### Test–retest reliability

Test–retest reliability indicates the size of measurement error between test and retest sessions. In addition to Intraclass correlation coefficient (ICC), the minimal detectable change (MDC) and standard error of measurement (SEM) are calculated. These parameters facilitate comparison and interpretation of data. Test–retest reliability of the numeric data was assessed by using the Intraclass Correlation Coefficient [ICC(1,1)], one-way random effects, absolute agreement, single rater/measurement. The values were classified as follows: < 0.5 indicate poor, 0.50–0.75 indicates moderate, 0.75–0.9 indicates good reliability, and 0.90 indicates excellent reliability^31^. The acceptable ICC values need to be equal to or greater than 0.70 for a clinically meaningful measurement^32^.

SEM indicates that the true value lies within ± 1SEM of measurement with 68% confidence and ± 2SEM with 95%. A limit of 5° of measurement error is set based on previous literature^24,33^ and (SEM) was calculated using SEM = $SD x \sqrt {1 - ICC}$ where ICC was from previous calculation^24^. In order to account for the extent of change that is not the consequence of random variation or measurement error, the minimum detectable change at the 95% confidence level (MDC_95_) was calculated using MDC_95_ = (2) × 1.96 × SEM^34^.

The goniometer, as a standard clinical measuring tool of angular joint position, can only measure the static angular position of the MCP joints. Therefore, only maximal flexion of the MCP joints will be evaluated in this paper as it reflects the functional range of motion and affects the size of object the hand can hold.

## Results

Ten healthy right-handed participants with a mean age of 34.6 years (eight men and two women) were evaluated with the air-guitar system and a finger goniometer (Table 1).

**Table 1:** Demographic data of asymptomatic participants recruited in the study.

	Mean ± SD	Minimal–maximal values
Age (year)	34.6 ± 12.2	22.0–55.0
Height (m)	1.7 ± 0.1	1.5–1.9
Weight (kg)	62.5 ± 10.6	47.0–83.0
BMI (kg/m^2^)	22.7 ± 4.9	19.1–31.6

N = 10, Male = 8 min–max.

BMI, Body mass index.

### Concurrent validity

**Fig. 5:**
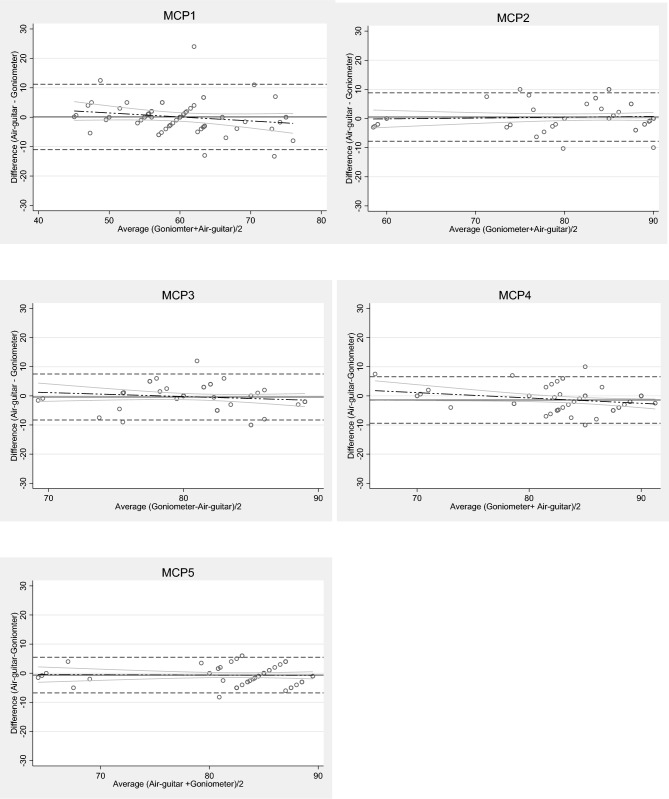
60 paired differences between flexion range of metacarpophalangeal (MCP) joints measured by goniometer and by air-guitar device. Solid line represents mean; upper dashed line shows the mean ± 1.96 standard deviation (SD) and lower dashed line shows the mean − 1.96SD. The long dash lines show the regression lines with gray continuous lines showing confidence interval limits.

According to Table 2, CCC of MCP3 is 0.62, indicating moderate agreement, while other MCP joints range from 0.8 to 0.9 indicating very strong agreement between measurements between the goniometer and the air-guitar system. The mean differences (close to zero) are less than 5°of the acceptable value priori set. The Bland–Altman plots show a graphical presentation of all 60 pairs of measurements taken from two sessions (Fig. 5). The zero line lies within the range of differences between the goniometer and the air-guitar system. The limit of agreement is ranging between 6 and 11° across MCP joints. Though the regression lines show small negative trend along the graph indicating small proportional error (Supplementary Table S1). However, a negative trend is expected when readings are close to the upper limit of 90°. The differences are very small within the limit of agreement. There seem to be no tendency of bias across these joints.

**Table 2:** Validity parameters between the goniometer and the air-guitar system in the same sessions.

Measurements	Lin’s Concordance correlation coefficient (CCC)	Mean difference (°)	Limit of agreement (LoA) (°)
MCP1	0.76	0	− 11, 11
MCP2	0.90	1	− 7, 8
MCP3	0.62	0	− 8, 8
MCP4	0.75	− 1	− 9, 7
MCP5	0.87	− 1	− 7, 6

MCP-metacarpophalangeal joint.

### Test–retest reliability

The maximal flexion of MCP joints^1–5^ were reported in Table 3. MCP angles between two methods were comparable. All ICC values from the goniometer and from the air-guitar are indicative of good to excellent reliability and well above the acceptable level of 0.7 (Table 4). However, the interval estimates (95% CI) of the air-guitar readings seem to be lower than those of the goniometer. Most MDC_95_ and SEM from the air-guitar measurements seem to be similar to the goniometer measurements. MDC_95_ and SEM from MCP1 and MCP2 appear to be slightly greater than those of others.

**Table 3:** Descriptive statistics for test -retest measurements from the goniometer and the air-guitar system.

Joints	Mean of maximum Flexion (SD) [Min–Max]							
	Goniometer				Air–guitar			
	Test		Retest		Test		Retest	
MCP1	61° (9°)	[47–75]	59° (8°)	[47–75]	60° (8°)	[45–73]	59° (7°)	[50–74]
MCP2	84° (9°)	[60–90]	80° (11°)	[60–90]	85° (10°)	[58–90.]	81° (10°)	[58–90]
MCP3	81° (5°)	[72–90]	81° (4°)	[77–90]	81° (5°)	[71–87]	80(4°)	[71–86]
MCP4	84° (5°)	[72–90]	85° (7°)	[68–90]	83° (5°)	[71–89]	83° (5°)	[70–89]
MCP5	83° (7°)	[65–88]	84° (6°)	[68–88]	82° (7°)	[64–89]	83° (6°)	[67–88]

MCP, metacarpophalangeal joint; SD, standard deviation.

**Table 4:** Test–retest reliability of the goniometer and the air-guitar system.

Measurements	Methods	ICC (95% CI)	SEM	MDC_95_
MCP1	Goniometer	0.84 (0.38–0.96)	3	9
	Air-guitar	0.82 (0.32–0.95)	3	9
MCP2	Goniometer	0.88 (0.54–0.97)	4	10
	Air-guitar	0.88 (0.55–0.97)	3	9
MCP3	Goniometer	0.76 (0.79–0.94)	2	6
	Air-guitar	0.93 (0.74–0.98)	1	3
MCP4	Goniometer	0.90 (0.62–0.97)	2	5
	Air-guitar	0.99 (0.96–1.00)	1	1
MCP5	Goniometer	0.97 (0.89–0.99)	1	3
	Air-guitar	0.97 (0.88–0.99)	1	3

MCP, metacarpophalangeal joint; ICC, intraclass correlation coefficient; CI, confidence interval; SEM, standard error of measurement; MDC_95_ minimum detectable change at the 95% confidence level.

## Discussion

### Concurrent validity

In this study, the goniometer is used as a reference method to demonstrate the validity of measurements of active flexion from the air-guitar for MCPs. Image techniques such as X-rays may provide more accurate angles of joints. However, the cost and risk may be inappropriate for this study, and the goniometer is a current clinical standard practice to monitor angles of joints. Therefore, the result of this study could facilitate the current clinical practice.

The CCC was selected to demonstrate the concurrent validity of MCP measurements between the goniometer and the air-guitar systems, yielding CCC values ranging from 0.62 to 0.90. This is considered very strong^26^. However, McBride’s criteria suggest that a CCC value of less than 0.90 indicates poor agreement, 0.90–0.95 indicates moderate agreement, 0.95–0.99 indicates substantial agreement, and greater than 0.99 indicates almost perfect agreement. By McBride’s criteria^35^, the results might be deemed very poor since the readings were not identical. However, McBride’s criteria may not be entirely appropriate for this study due to the differing resolutions of the devices: the goniometer has a resolution of 5°, whereas the air-guitar system has a resolution of 0.01°. This discrepancy in resolution likely accounts for the observed differences between the two methods.

The mean differences between the two methods (approximately 1°) are well within the acceptable range (5°), supporting the validity of the air-guitar system for assessing active flexion of the MCP joints. However, the LOA ranges from 6° to 11°, suggesting that caution should be exercised when interpreting data obtained with the air-guitar system. This wide LOA range may be attributed to the differing resolutions of the devices, as previously mentioned. Additionally, measurements were taken at the end of active flexion, and despite identical verbal instructions, some variations within sessions may have occurred. Our results suggest that there is an absence of systematic errors as the mean differences between the two measurements are consistently close to zero and within the limits of agreement. As well as the proportional errors indicated by the regression lines and the confidence interval limits are relatively small within the limit of agreement. Moreover, there is no tendency for one measurement to be consistently higher or lower than the other, across different values of readings. Although the measurements from the two methods are not identical, the results indicate a very strong agreement between them. While the air-guitar system may not be a suitable replacement for the goniometer in clinical settings, it serves as a useful device for monitoring the active maximal flexion of the MCP joints during home-based training. Future research should focus on validating the dynamic measurements of the air-guitar device against gold standards, such as 3D motion analysis systems, to accurately quantify dynamic data.

### Test–retest reliability

The test–retest reliability of MCP flexion measurements using the air-guitar system proved acceptable for clinical use, with ICCs exceeding 0.7^32^. Both measurement methods demonstrated similarly high ICCs, with the air-guitar system showing smaller CIs for MCP1 and MCP3-MCP5, indicating less variability compared to the goniometer. This may be because the resolution of the Air-guitar (0.01°) is finer than that of the Jamar finger goniometer (5°), and the use of the device may reduce some human errors during test–retest measurements. The current goniometer technique involves several placements of the flat goniometer arms on the fingers and hand surfaces, which are not completely flattened. This difficulty is similar to the wrist, as suggested by Armstrong et al.^36^. Also, these two joints (MCP4-5) are relatively difficult to approach manually. In contrast, the goniometer measurement of the MCP1 showed slightly higher ICC and narrower range of CI than those from the air-guitar system, this may be because of stabilization of the surrounding joints. The manual approach required the examiner to stabilize the carpometacarpal joints while taking measurements, while the air-guitar system did not. Moreover, with the goniometer and therapist’s hand, the movements in the different planes were well stabilized. Whereas the patient actively moved without stabilization when using the air-guitar system. The test–retest reliability of the air-guitar is comparable to that of the goniometer and likely to be able to detect changes of MCP flexion over time course with caution of the MCP1-2 joints.

The SEM and MDC_95_ calculated in this study were well below the 5° limit established by clinical routine measurements using a goniometer. The SEM and MDC_95_ reflect measurement errors in the same units as the measurements themselves and are valuable for monitoring progress over time. SEM values for all joints measured by the air-guitar system ranged between 1° and 3°, similar to those from the goniometer. In our data, the MDC_95_ for the air-guitar system ranged from 1° to 9°, and for the goniometer from 3° to 10°, indicating comparable results. This suggests that the air-guitar system is as capable of detecting changes in flexion as the goniometer.

Regarding goniometer measurements, the goniometer readings across all MCPs were roughly the same range of the previous reports. The MDC_95_ range of 3°–10° were between 19° and 24°as previously reported by Reissner et al. and the SEM range of 1°–4° were between 7° and 9° reported by Reissner et al.^24^. The ICC from our goniometer measurements ranging from 0.75 to 0.97 was also within the ranges (0.59–0.86)^24^ and (0.68–0.93)^37^, as previously reported. These ensure the goniometer data quality in this protocol is comparable to the others.

## Limitation

This study is done as a preliminary study in a small sample size to demonstrate the concurrent validity and test–retest reliability of the hand therapeutic device for measurement of the maximal MCP flexion, approximately 90°^38^, as a monitoring device for a home-based therapeutic exercise. The air-guitar system is validated using the clinical finger goniometer which is usually small with increments of 5°. Compared with 0.01° increment of the air-guitar, 5° is large. However, since the goniometer is the gold standard for measuring range of motion in clinical practice, the air-guitar is compared to it.

Blinding of measurements between morning and afternoon sessions for the same device could not be implemented, as the same clinician and technician conducted both sessions to ensure consistent measuring conditions. To minimize potential bias, measurements were recorded on separate blank case record forms, preventing the clinician and reader from seeing previous readings. Additionally, each participant had 15 measurements, and with two to three participants per morning session, it was unlikely that the clinician and technicians would remember previous readings and influence the results.

The concurrent validity and test–retest reliability shown in this study seem to be acceptable for monitoring joint range in healthy participants. However, the minimal clinical change in different patient groups where the device may be clinically used should be further investigated to facilitate clinical application. In addition, the concurrent validity and test–retest reliability are limited to static maximal flexion measurements only. The maximal flexion may strongly associate with the functional flexion [MCP is ranging from 19 to 71°^39^] so it can only be used with caution. The functional flexion of the hand in different postures and movements can be measured using the air-guitar system. To quantify these dynamic movements and ensure certainty of use, it requires further investigation against 3D motion analysis as a gold standard.

## Conclusion

As a home-based therapeutic device, the air-guitar monitoring characteristics could facilitate monitoring of MCP change over a time course with acceptable test–retest reliability as a clinically meaningful measurement tool and comparable to the goniometer with less than 5° difference.

### Supplementary Information

Supplementary Table S1.

Supplementary Information.

## Data Availability

The data that support the findings of this study are not openly available due to reasons of sensitivity and are available from the corresponding author upon reasonable request.
