# Supplementary material for: Validity and reliability of a finger training tool for assessing metacarpal phalangeal joint ranges of motion in asymptomatic participants
Source: Sci Rep. 2024 Aug 29;14:20113. doi: 10.1038/s41598-024-71094-y (PMC11362323; doi:10.1038/s41598-024-71094-y)
Supplement: Supplementary file 1 — Supplementary Table S1. [file 41598_2024_71094_MOESM1_ESM.docx]

Supplementary file 1

Table S.1 Regression equations across metacarpophalangeal joints

| Joints | Regression line with confidence interval limits |
| --- | --- |
| MCP1 | y= -0.14(-0.33 to 0.05)x +8.23(-3.26 to 19.7) |
| MCP2 | y= 0.03(-0.09 to 0.15)x -1.77(-11.62 to 8.08) |
| MCP3 | y= -0.13(-0.39 to 0.12)x +10.60(-9.81 to 31.00) |
| MCP4 | y= -0.18(-0.37 to 0)x+ 13.90(-1.50 to 29.30) |
| MCP5 | y= -0.01(-0.15 to 0.13)x +0.15(-11.13 to 11.44) |
