# Supplementary material for: Validity and reliability of a finger training tool for assessing metacarpal phalangeal joint ranges of motion in asymptomatic participants
Source: Sci Rep. 2024 Aug 29;14:20113. doi: 10.1038/s41598-024-71094-y (PMC11362323; doi:10.1038/s41598-024-71094-y)
Supplement: Supplementary file 2 — Supplementary Information. [file 41598_2024_71094_MOESM2_ESM.docx]

# Research protocol:

**Air Guitar: Tele-rehabilitation system: tracking physical changes, tracking hand therapy and analyzing movements for stroke survivors**

## Project summary

Rationale: 80 of stroke survivors are likely to experience muscle abnormalities that affect neural functions in areas such as the face, arms, and legs. They require long term rehabilitation of the affected area. Upper limb rehabilitation involves specific task-oriented movements and high-intensity training. This approach leads to changes in corticomotor outputs, which are associated with hand function, especially during the first six months when the brain's recovery rate is highest. Therefore, continuous rehabilitation from hospital to home is paramount. In response to this need, our research team has developed a prototype remote hand function rehabilitation system using technology to assess and monitor physical parameters such as range of motion and grip strength. The system includes structured training programs and progress assessments to tailor rehabilitation plans to individual patients. This approach ensures the efficient transmission of valuable data to rehabilitation professionals, enhancing patient care and outcomes.

### Objectives:

1. Develop a telerehabilitation system which can be used at home for upper limb rehabilitation

2. Test the prototypes and its components: range of motion, grip strength, software for hand and finger movement training.

Methods: 1. Develop a telerehabilitation system which can be used at home for upper limb rehabilitation based on requirements from the patients and the occupational therapists.

2. tests the components of system: validity, repeatability of the range of motion and grip strength

3. Tests the usability of the software.

Populations: Healthy participants

Time frame: 1 year

Expected outcomes: a prototype system is developed with validated ROM and grip strength measurements of intraclass correlation coefficient (ICC) value of 0.7. This is the minimum acceptable threshold for evaluating measurement tools used in clinical settings. Also the software is tested for usability.

## General information

Air Guitar: Tele-rehabilitation system: tracking physical changes, tracking hand therapy and analyzing movements for stroke survivors

STUDY CODE: REH-2565-08821 Research ID: 8821

**Name and address of the sponsor/funder.**

MED CMU Health Innovation Center (MedCHIC)

Faculty of Medicine, Chiang Mai University (Grant number 356/2022)

110 Intavaroros Rd., Amphoe Mueang, Chiang Mai, Thailand

50200

**Principal investigator**

Miss Kanyaluck Utarachon

Address: Department of Physical Rehabilitation Medicine, Faculty of Medicine, Chiang Mai University Chiang Mai, Thailand

e-mail address: maam130120@gmail.com

Telephone: 053-935547.

Fax: 053-936322

Roles: Investigation, Writing, Review and editing

**Investigators**

| **Consultant** | | | | **Roles** |
| --- | --- | --- | --- | --- |
|  | Name-surename | Title | Address and telephone number |  |
| 1 | Jakkrit Klaphajone | Associate Professor, Dr. (MD.) | Department of Physical Rehabilitation Medicine, Faculty of Medicine, Chiang Mai University Chiang Mai, Thailand | Conceptualization, Validation, Writing |
| 2 | Pisith Singjai | Associate Professor, Dr.(PhD) | Department of Physics and Materials Science, Faculty of Science, Chiang Mai University Chiang Mai, Thailand | Conceptualization, Writing |
| 3 | Pakorn Wiwatwongwana | Assistant Professor. Dr.(MD) | Department of Physical Rehabilitation Medicine, Faculty of Medicine, Chiang Mai University Chiang Mai, Thailand | Conceptualization, Writing |
| **Investigators** | | | |  |
| 4 | Teesasil Kumpica | Dr.(Phd) | Department of Physical Rehabilitation Medicine, Faculty of Medicine, Chiang Mai University Chiang Mai, Thailand | Conceptualization, Investigation, Writing |
| 5 | Nuanlaor Thawinchai | Associate Professor. Dr.(PhD) | Department of Physical Therapy, Faculty of Associated Medical Sciences, Chiang Mai University Chiang Mai, Thailand | Conceptualization Validation, Writing |
| 6 | Teerawat Kamnerdsiri | Dr.(Phd) | College of Arts, Media, and Technology. Chiang Mai University. Chiang Mai University Chiang Mai, Thailand | Investigation, Writing |
| 7 | Pornsuree Kuvijitsuwan | Dr.(Phd) | Department of Physical Rehabilitation Medicine, Faculty of Medicine, Chiang Mai University Chiang Mai, Thailand | Conceptualization, Validation, Investigation, Writing |

## Rationale & background information

Stroke is a highly prevalent non-communicable disease and a major global public health issue. In Thailand, reports from the Policy and Strategy Bureau of the Ministry of Public Health indicate that the number of stroke patients increased from 293,463 in 2016 to 304,807 in 2017, and to 331,086 in 2018. Specifically, there were 7,948 stroke patients in Chiang Mai province. Among these patients, 80% who survive experience muscle abnormalities that affect neural functions in areas such as the face, arms, and legs.(1)

Effective rehabilitation for patients with arm weakness due to neurological conditions like stroke involves specific task-oriented movements and high-intensity training. (1) This approach leads to changes in corticomotor outputs, which are associated with hand function, especially during the first six months when the brain's recovery rate is highest. (2) Current rehabilitation techniques include Bobath, the Motor Relearning Programme, and mirror therapy (3),(4) However, reports indicate that 70-80% of stroke survivors who return home still face limitations in their daily activities despite undergoing rehabilitation. (5)

After being discharged from the hospital, patients need to continue their training, either as outpatients receiving close supervision from a rehabilitation team or independently at home, particularly during the first six months of optimal recovery. Outpatient rehabilitation typically involves 10-session courses, costing approximately 2,000 THB per session for travel, food, and accommodation. Patients may visit the hospital 1-3 times per week, depending on availability. Surveys have shown that both patients and caregivers face difficulties in accessing rehabilitation advice and using assistive devices correctly, which may pose risks due to the insufficient number of rehabilitation professionals.(6) Prolonged rehabilitation sessions at the hospital impose additional financial burdens on patients, who often lack motivation to adhere to long-term conventional rehabilitation programs. Consequently, many patients do not follow rehabilitation guidelines strictly and completely. To enable patients to maximize their hand function and self-care abilities, they need adequate and effective hand rehabilitation from specialists, along with motivational support.

In response to this need, our research team has developed a prototype remote hand function rehabilitation system using technology to assess and monitor physical parameters such as range of motion and grip strength. The system includes structured training programs and progress assessments to tailor rehabilitation plans to individual patients. This approach ensures the efficient transmission of valuable data to rehabilitation professionals, enhancing patient care and outcomes.

## Study goals and objectives

1. Develop a telerehabilitation system which can be used at home for upper limb rehabilitation

2. Test the prototypes and its components: range of motion, grip strength, software for hand and finger movement training.

## Study design

• Pilot study, Research and development of medical devices (Proof of concept/pre -clinical stage)

• Target population: Healthy participants to test performance of the prototypes

## Methodology

### Equipment and materials

Prototype: air-guitar system

Finger Goniometer for measuring range of motions: metacarpal phalangeal joints (MCP): 1,2,3,4,5

Dynamometer for assessment of grips strength

Pinch gauge for assessment of pinch strength

Optoelectronic system (BTS Bioengineering, Milan, Italy) with 46 9 -mm markers

Questionnaires: System Usability Scale (SUS) Thai version (7, 8)

### Procedures

#### Lab tests for the air-guitar system

10 Healthy participants involved in this study. After signing a consent form, general information will be collected. The participant will be randomly assigned to assess MCP ROM using a finger Jamar goniometer or the air-system first. The order is computer-generated. The participant sits with appropriate posture, appropriate chair height of chair and table. His elbow should 90° flexion on the table.

Place the right hand flat on the table and measure the range of motion of the fingers. Stabilize the joints adjacent to the one being measured and ask the participant to move their fingers: the thumb (flexion-extension, abduction-adduction) and the other fingers (flexion-extension). Measure the initial and maximum movement values without moving the adjacent joints using a goniometer according to standard clinical procedures. Record the measurements three times and report the average value.

**1. Range of motion**

**1.1 Metacarpophalangeal Joint (MCP) of the Fingers (1-5): (9, 10)**

Starting Position: Position the upper forearm in full supination.

Goniometer Placement: Place the fulcrum on the dorsal aspect of the MCP joint. Align one arm of the goniometer along the midline of the metacarpal bone and the other along the proximal phalanx.

Flexion Measurement: Ask the participant to flex their finger maximally and record the full flexion angle. Repeat this measurement three times.

Extension Measurement: Return the finger to the neutral position and ask the participant to extend their finger maximally, recording the full extension angle. Repeat this measurement three times. (Extension is measured as the return movement to the starting position).

**1.2 Carpometacarpal Joint of the Thumb (CMC1):**

Starting Position: Begin with the thumb in a neutral position.

Goniometer Placement: Place the fulcrum on the palmar aspect of the CMC1 joint. Align one arm of the goniometer along the midline of the radius and the other along the first metacarpal.

Initial Reading: Record the initial angle,

Flexion Measurement: Ask the participant to flex their thumb maximally and record the full flexion angle (noting the change from the initial reading). Repeat this measurement three times.

Extension Measurement: Return the thumb to the neutral position. Then, ask the participant to extend their thumb maximally and record the full extension angle (noting the change from the initial reading). Repeat this measurement three times.

**1.3. Abduction Measurement of the CMC1:**

Starting Position: Place the lower forearm in a position midway between supination and pronation.

Goniometer Placement: Position the fulcrum on the lateral aspect of the radial styloid process. Align one arm of the goniometer along the midline of the first and second metacarpals.

Measurement: Record the angle of abduction. (Adduction is measured as the return movement to the starting position).

Table 1 Goniometer measurement of healthy participants

| **Joint** | **Movement** | **Range of motion (°)** |
| --- | --- | --- |
| *Fingers* |  |  |
| MCP joints | flexion | 90-100 |
|  | extension | 20-45 |
|  | Abduction/adduction | 25 |
| *Thumb* |  |  |
| CMC joints | flexion | 15-25 |
|  | extension | 15-35 |
|  | Abduction/adduction | 40-50 |
| MCP joints | Flexion/extension | 50-60 |

Table 2 Functional range of motion

Mean angle of flexion for fingers and thumb at completion of prehension activities in degrees

| **Activity** | **Goniometer** | | | | |
| --- | --- | --- | --- | --- | --- |
|  | **Finger** | | | **Thumb** | |
|  | MCP joint | PIP joint | DIP joint | MCP joint | IP joint |
| Tip pinch | 58 | 76 | 33 | 22 | 25 |
| Palmar(pad)pinch | 35 | 40 | 50 |  |  |
| Lateral (key) pinch | 62 | 76 | 46 | 20 | 16 |
| Power grip (Hume) | 72 | 78 | 50 | 23 | 36 |

**2.Strength test measurement**

*2.1 Hydraulic dynamometer(11)*

**1. Participant Positioning:** Ask the participant to sit comfortably with their back against the chair. Ensure the elbow is flexed at 90 degrees and positioned at their side, with feet flat on the floor. If necessary, request the participant to roll up their pant legs to allow for continuous observation of muscle contractions during the squeezing task.

**2.**Demonstrate how to hold and squeeze the dynamometer, ensuring the participant understands the proper technique. The participant should perform the task in a way that feels comfortable and without any resistance. The initial reading on the device should be 0. Adjust the grip distance of the dynamometer to fit the participant's hand size appropriately.

3. The researcher may support the dynamometer with their hand, ensuring it does not restrict its movement.

4. Instruct the participant to squeeze the dynamometer with maximum force and for as long as possible. Provide verbal encouragement such as, “Squeeze... harder, harder... stop squeezing.” Record the reading, allow the participant to rest, and repeat the procedure three times when they are ready.

Table 3 Normative grip strength data


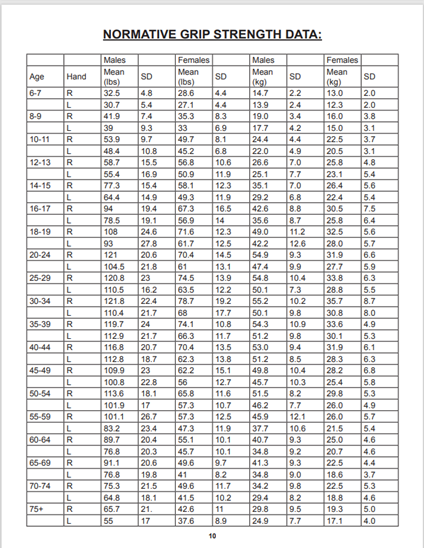


Source Instruction manual for Hydraulic dynamometer

*2.2 Hydraulic Pinch Gauge Measurement:*

1. Participant Positioning:

Ask the participant to sit comfortably with their back against the chair, elbow flexed at 90 degrees, and positioned at their side, with feet flat on the floor. If necessary, request the participant to roll up their pant legs to allow for continuous observation of muscle contractions during the squeezing task.

2. Researcher Positioning and Instruction:

The researcher should sit opposite or beside the participant to help support the pinch gauge and provide encouragement. Instruct the participant to squeeze the pinch gauge with maximum force and for as long as possible, using verbal encouragement such as, “Squeeze... harder, harder... stop squeezing.” Record the reading, allow the participant to rest, and repeat the procedure three times when they are ready.

3. Pinch Strength Measurement:

Tip Pinch: With the hand pronated, place the pinch gauge between the thumb and the finger being tested.

Palmar Pinch: With the hand pronated, place the pinch gauge between the thumb and the index or middle finger.

Lateral Pinch: With the palm facing inward, place the pinch gauge between the thumb and the proximal interphalangeal joint of the index finger.

Table 4 Normative tip pinch strength

| Tip pinch Strength (kg) | | | | | | | | |
| --- | --- | --- | --- | --- | --- | --- | --- | --- |
|  | Male | | | | Female | | | |
|  | Dominance | | Non dominance | | Dominance | | Non dominance | |
| Age | Mean | SD | Mean | SD | Mean | SD | Mean | SD |
| 22-24 | 8.1 | 1.4 | 7.7 | 1.0 | 5.0 | 0.9 | 4.7 | 0.8 |
| 25-29 | 8.2 | 2.0 | 7.9 | 2.3 | 5.4 | 0.8 | 5.1 | 0.8 |
| 30-34 | 7.8 | 3.0 | 7.9 | 2.2 | 5.7 | 1.4 | 5.3 | 1.3 |
| 35-39 | 8.1 | 1.6 | 8.0 | 1.7 | 5.2 | 1.1 | 5.4 | 1.1 |
| 40-44 | 8.0 | 1.8 | 8.0 | 1.6 | 5.2 | 1.2 | 5.0 | 1.4 |
| 45-49 | 8.4 | 2.2 | 7.9 | 1.8 | 5.9 | 1.4 | 5.4 | 1.2 |
| 50-54 | 8.2 | 1.8 | 8.0 | 1.8 | 5.6 | 1.0 | 5.1 | 1.1 |
| 55-59 | 7.5 | 1.5 | 6.8 | 1.7 | 5.3 | 0.8 | 4.7 | 0.6 |
| 60-64 | 7.1 | 1.8 | 6.9 | 1.7 | 4.5 | 0.9 | 4.5 | 0.9 |
| 65-69 | 7.7 | 1.9 | 6.9 | 1.3 | 4.8 | 0.9 | 4.7 | 1.1 |
| 70-75 | 6.2 | 1.2 | 6.0 | 1.2 | 4.5 | 1.2 | 4.4 | 1.0 |
| >75 | 6.3 | 1.5 | 6.3 | 1.7 | 4.3 | 1.3 | 4.2 | 1.1 |

Source Instruction manual for Pinch gauge (Convert: pounds to kg)

Table 5 Normative palmer pinch strength

| **Palmer pinch Strength (kg)** | | | | | | | | |
| --- | --- | --- | --- | --- | --- | --- | --- | --- |
|  | Male | | | | Female | | | |
|  | Dominance | | Non dominance | | Dominance | | Non dominance | |
| Age | Mean | SD | Mean | SD | Mean | SD | Mean | SD |
| 22-24 | 12.0 | 2.4 | 11.6 | 2.6 | 7.7 | 1.0 | 7.3 | 1.3 |
| 25-29 | 11.7 | 1.9 | 11.3 | 1.9 | 8.0 | 1.4 | 7.7 | 1.4 |
| 30-34 | 11.1 | 2.1 | 11.4 | 2.6 | 8.7 | 2.3 | 8.1 | 2.2 |
| 35-39 | 11.8 | 1.8 | 11.7 | 2.4 | 7.9 | 1.9 | 7.7 | 1.5 |
| 40-44 | 11.0 | 1.9 | 11.2 | 2.2 | 7.7 | 1.4 | 7.5 | 1.6 |
| 45-49 | 10.8 | 1.5 | 10.7 | 1.7 | 8.1 | 1.4 | 7.9 | 1.3 |
| 50-54 | 10.7 | 2.4 | 10.8 | 2.6 | 7.8 | 1.4 | 7.4 | 1.3 |
| 55-59 | 10.7 | 2.2 | 9.6 | 2.0 | 7.2 | 1.4 | 7.4 | 1.3 |
| 60-64 | 9.8 | 1.5 | 9.5 | 1.4 | 6.7 | 1.4 | 6.4 | 1.2 |
| 65-69 | 9.6 | 1.4 | 9.5 | 1.4 | 6.4 | 1.4 | 6.2 | 1.5 |
| 70-75 | 8.1 | 1.5 | 8.5 | 1.5 | 6.5 | 1.2 | 6.3 | 0.9 |
| >75 | 8.4 | 1.9 | 8.2 | 1.7 | 5.4 | 1.2 | 5.2 | 1.2 |

Source Instruction manual for Pinch gauge (convert from pounds to kg)

Table 6 Normative lateral pinch strength

| **Lateral pinch Strength (kg)** | | | | | | | | |
| --- | --- | --- | --- | --- | --- | --- | --- | --- |
|  | Male | | | | Female | | | |
|  | Dominance | | Non dominance | | Dominance | | Non dominance | |
| Age | Mean | SD | Mean | SD | Mean | SD | Mean | SD |
| 22-24 | 11.7 | 1.6 | 11.2 | 1.5 | 7.9 | 0.9 | 7.3 | 0.9 |
| 25-29 | 12.0 | 2.2 | 11.3 | 2.1 | 8.0 | 0.9 | 7.5 | 0.9 |
| 30-34 | 11.9 | 2.2 | 11.8 | 2.3 | 8.4 | 1.4 | 8.0 | 1.6 |
| 35-39 | 11.7 | 1.4 | 11.8 | 2.3 | 8.4 | 1.4 | 8.0 | 1.6 |
| 40-44 | 11.5 | 1.2 | 11.3 | 1.8 | 7.5 | 1.4 | 7.1 | 1.4 |
| 45-49 | 11.6 | 1.8 | 11.2 | 2.0 | 7.9 | 1.4 | 7.5 | 1.3 |
| 50-54 | 12.0 | 2.0 | 11.7 | 1.9 | 7.5 | 1.1 | 7.2 | 1.2 |
| 55-59 | 10.9 | 1.9 | 10.4 | 2.1 | 7.1 | 1.1 | 6.6 | 1.0 |
| 60-64 | 10.4 | 2.4 | 10.0 | 1.8 | 7.0 | 1.2 | 6.3 | 1.1 |
| 65-69 | 10.5 | 1.8 | 9.9 | 1.6 | 6.8 | 1.2 | 6.4 | 1.3 |
| 70-75 | 8.7 | 1.1 | 8.6 | 1.4 | 6.5 | 1.3 | 6.2 | 1.4 |
| >75 | 9.2 | 2.1 | 8.6 | 1.4 | 5.7 | 1.0 | 5.1 | 1.2 |

Source Instruction manual for Pinch gauge (convert from pounds to kg)

**3. Measurement using the air-guitar system:**

a. Static measurement: Equip the participant's right hand with the air guitar hand device. Place the hand flat on the table and measure the range of motion of the fingers: thumb (circumduction, flexion-extension, abduction-adduction) and other fingers (flexion-extension). Record the data displayed on the air guitar system screen three times and report the average value. Next, instruct the participant to perform Tip Pinch, Palm Pinch, and Lateral Pinch movements, each repeated three times. Remove the device and record the movement (degrees) and grip strength (kg) data using the air guitar hand device.

Note: This study is a pilot study of the air guitar hand device prototype, so normative data is not yet available.

b. Dynamic measurement: Data Collection Using the Optoelectronic System:

Attach 46 double-sided tape markers to the specified positions on the participant's body as shown in Figure 1.


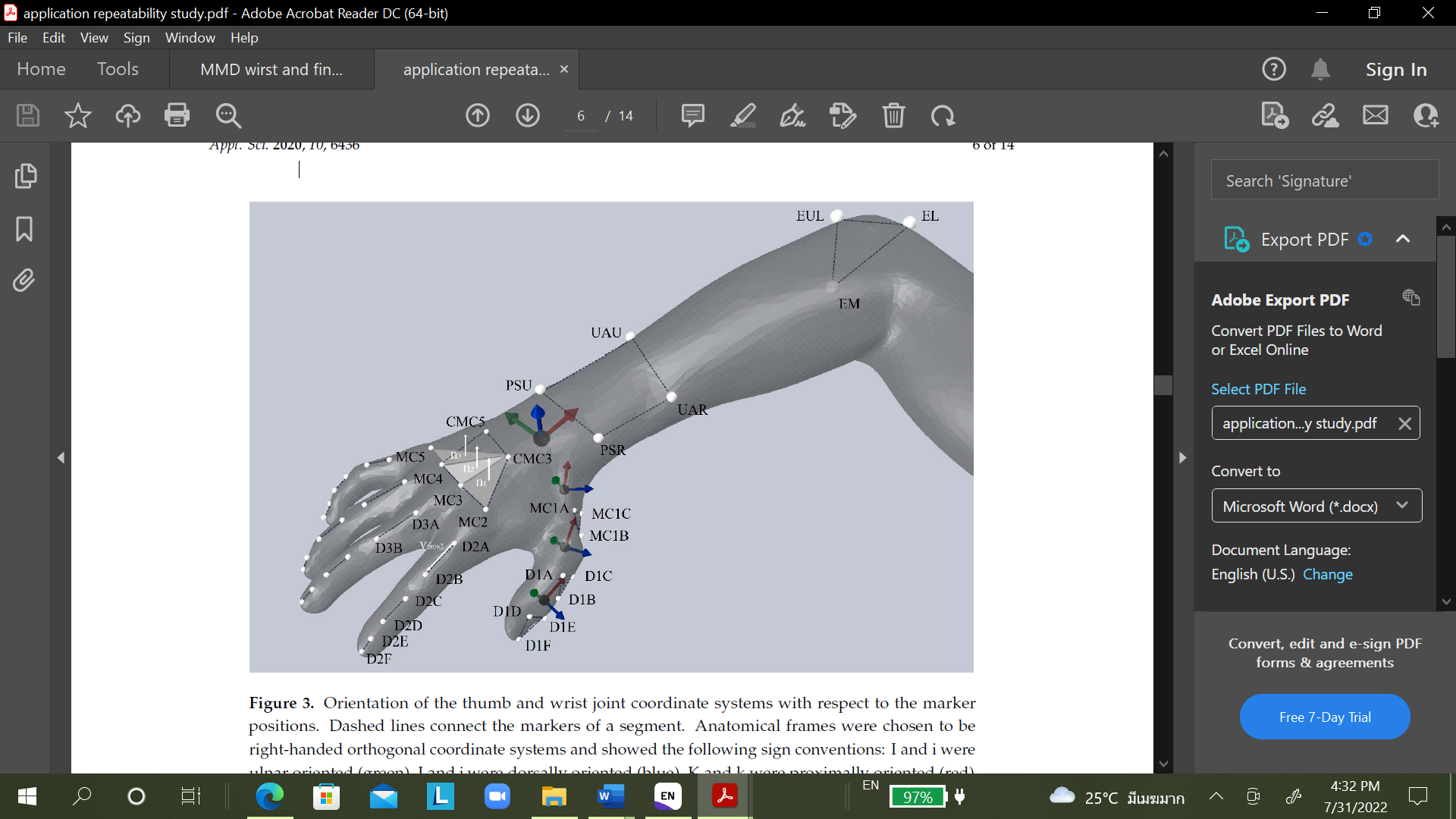


Figure 1 marker positions (12, 13)

(a)
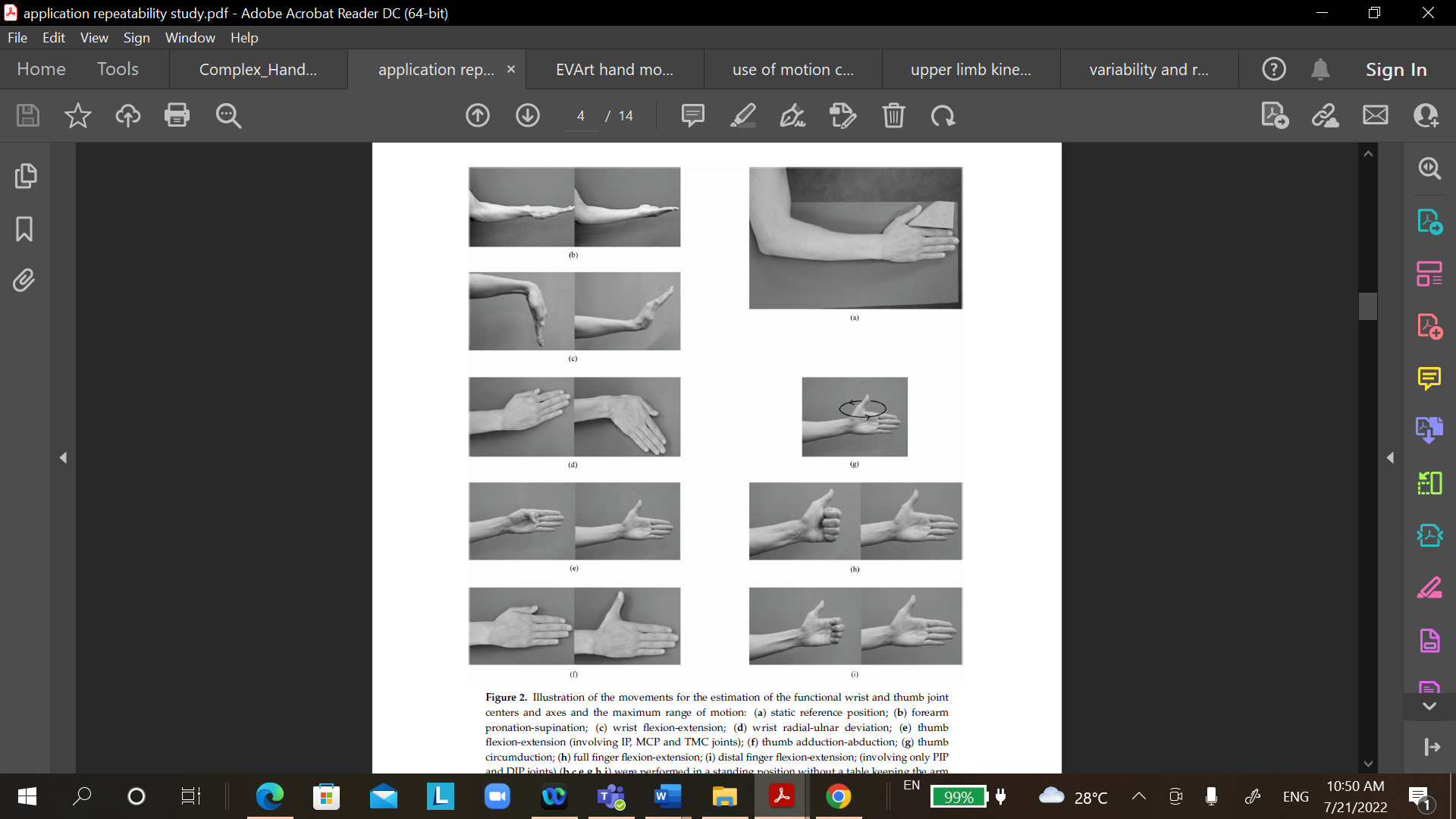
 (b)
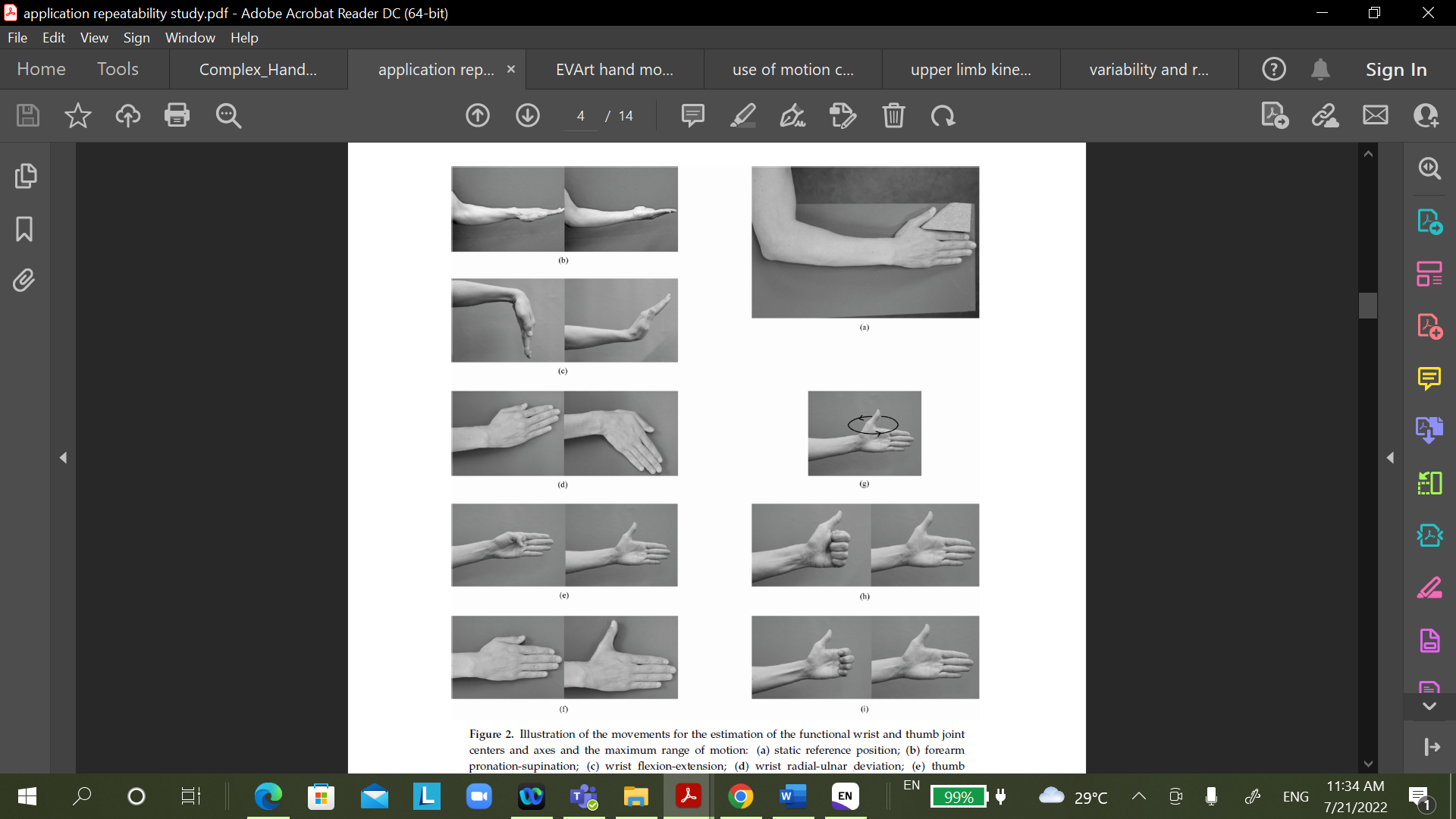


(c)
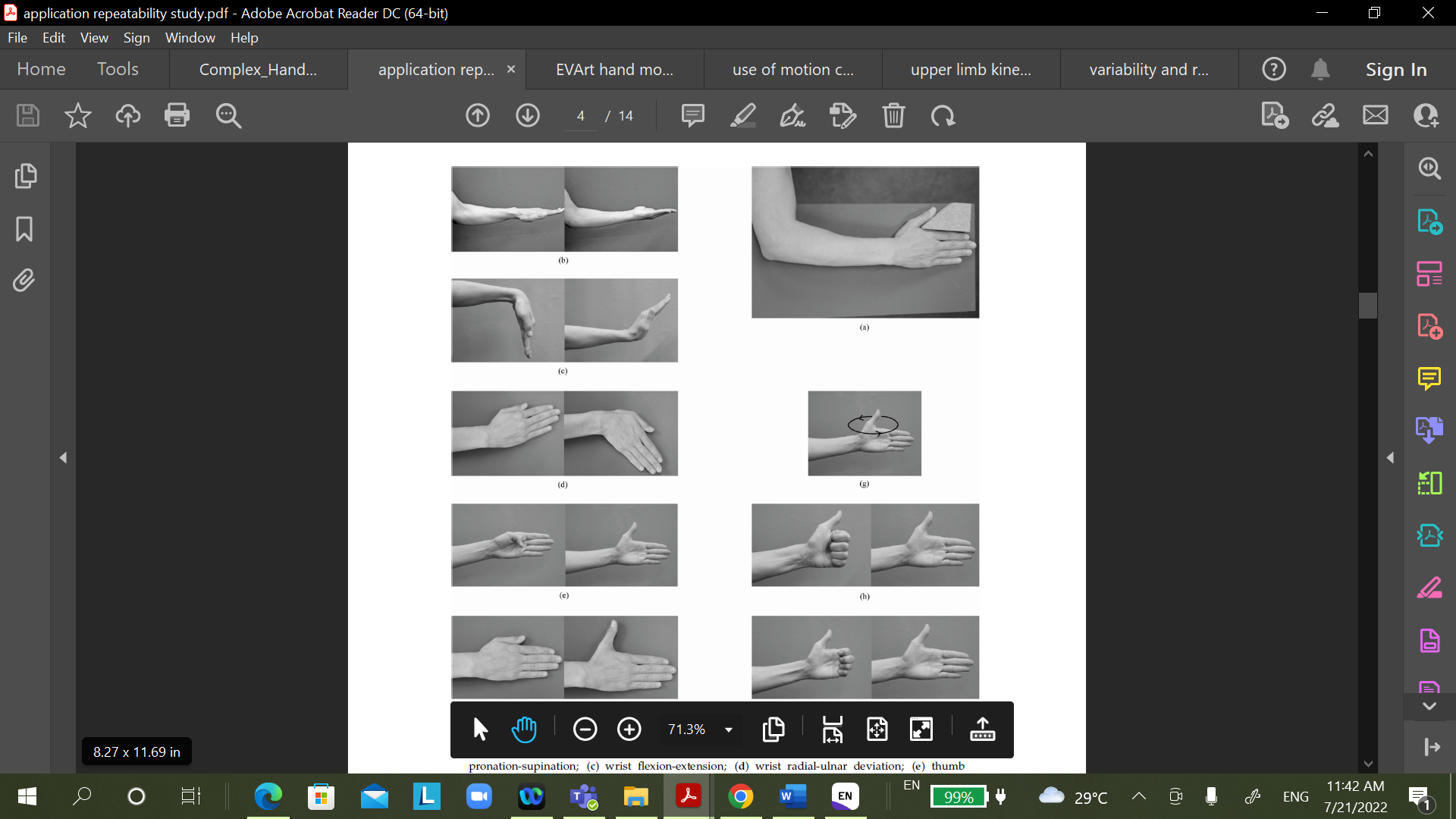
 (d)
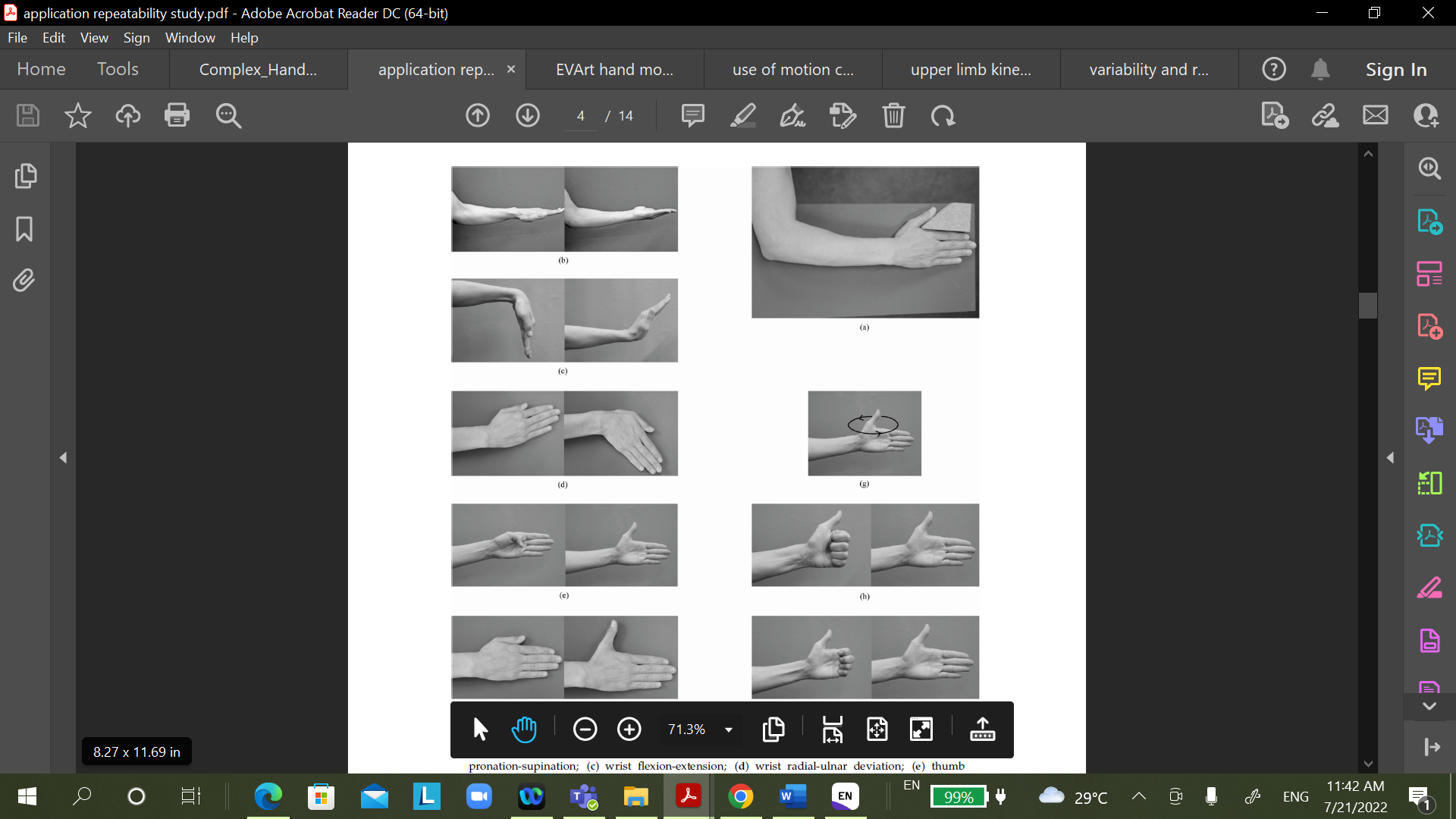
 (e)
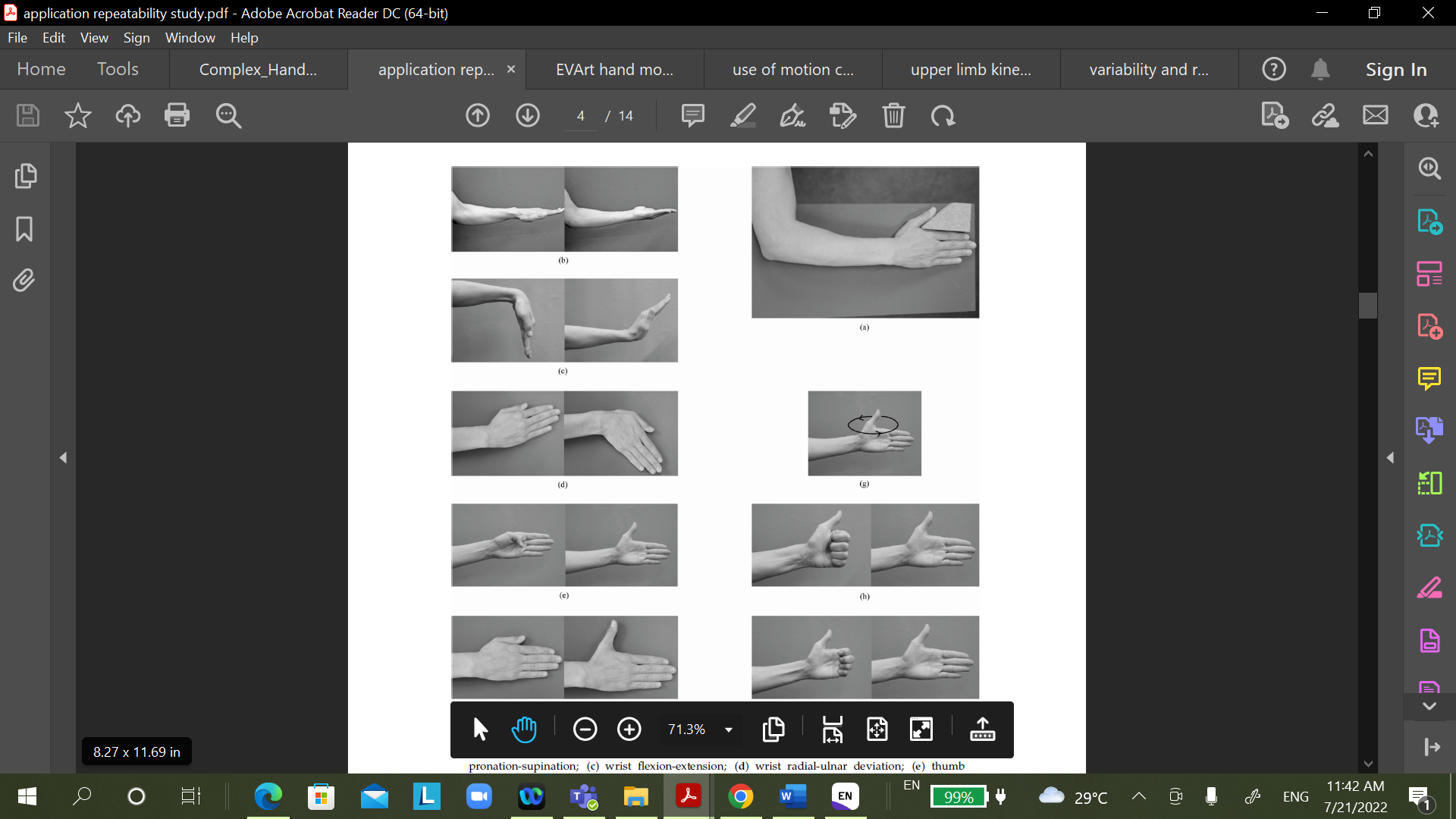


(f)
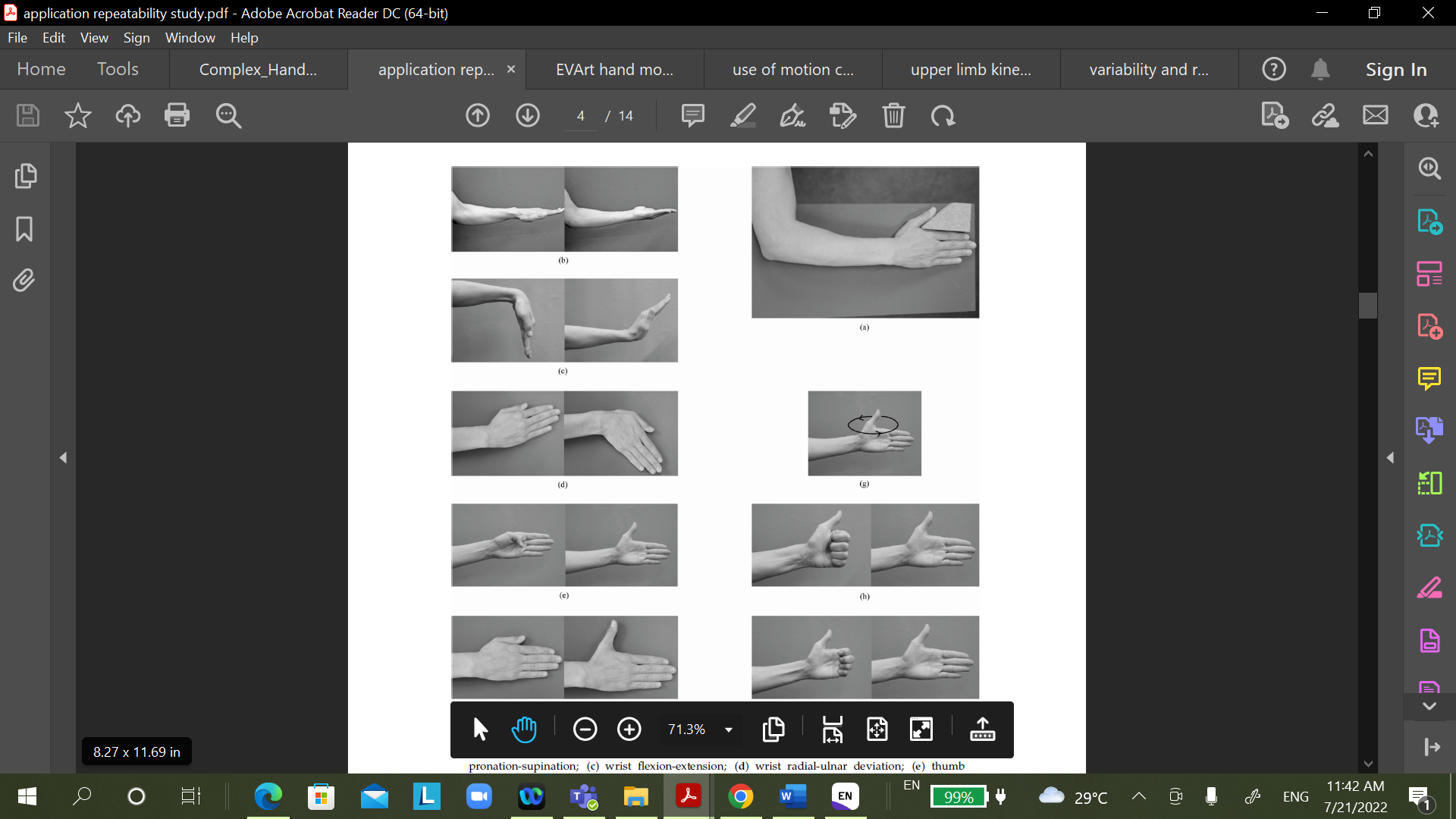


(a) Static reference position, (b) Thumb flexion-extension (involving IP, MCP and TMC joints, (c) Thumb adduction-abduction, (d) Thumb circumduction, (e)full finger flexion-extension, (f) distal finger flexion-extension (Fischer et al, 2019)

Figure 2 movements (13)

Move according to the specified motions above, including Tip Pinch, Palm Pinch, and Lateral Pinch, three times each, and then remove the device. Repeat Step ‘a-b’ to test test-retest reliability in the morning and afternoon of the same day for all three methods.

**4. Usability Assessment:**

Evaluate the ease of use of the gaming system and application using the Thai version of the System Usability Scale (SUS), a widely recognized tool. Collect data after the participant has used the system for 45 minutes in a single session.

### Outcome measures/ endpoints

Range of motion of the metacarpal phalangeal joints in flexion/extension, abduction/adduction

Grip strength/pinch strengths

System Usability Scale (SUS) Thai version

Flow of data collection

...............................................................

### Safety considerations

Risks of participants as a result of participation in a research study

This research project presents minimal physical risk to participants. Participants will wear a prototype air guitar hand device manufactured in a certified facility.

a. Electrical Risks:

There is a low risk of electric leakage or shock from using the air guitar hand device, as it operates on very low electricity and is produced in a certified facility. However, please adhere to the following guidelines:

Do not use any cables other than those provided, and do not use the device for purposes other than those specified.

The device is not waterproof, so avoid any contact with water to prevent electrical damage.

Do not touch the device or any medical equipment with wet hands or body.

Do not use the device if it is damaged or broken. If you find the device in an unsatisfactory condition, please notify the researcher immediately.

b. Risks from Motion Analysis System:

The use of double-sided adhesive markers in the three-dimensional motion analysis system may cause mild skin irritation, which typically resolves on its own.

c. Risks from Finger Movement Training:

The finger movement therapy used in this study is a standard hospital procedure and may cause temporary fatigue, which usually resolves on its own.

Participants will travel to the research site for scheduled appointments under this research project. Therefore, the project provides travel compensation of 300 THB.

This research project presents minimal physical risk to participants. Participants will wear a prototype air guitar hand device manufactured in a certified facility. During the three-dimensional motion data collection process, medical-grade double-sided adhesive tape will be used, which is safe. However, the skin where the tape is applied may exhibit redness, which typically resolves within 2-3 hours. In the event of any adverse reactions, participants should immediately inform the researcher and seek medical attention. The researcher will provide close monitoring and care.

### Follow-up

The researcher plans to inform the study participants of the research results via email and phone. The findings will be published without revealing the identities of the participants. Subsequently, the researcher will begin planning tests with a group of stroke patients.

### Data management and statistical analysis

1. Range of Motion and Strength from Physical Assessment and Training:

Descriptive statistics were used to describe the characteristics of the sample group. The means and standard deviations of the angle measurements (flexion/extension, abduction/adduction) of the metacarpophalangeal (MCP) joints of the fingers were obtained using three different systems. Grip strength was measured using a dynamometer, pinch gauge, and the air guitar hand device. Statistical analysis was performed to test the differences among the three systems using ANOVA or the Kruskal-Wallis test, depending on data distribution.

2. Test-Retest Reliability:

Reliability and variability of the range of motion and strength measurements were reported using the Intraclass Correlation Coefficient (ICC) and Standard Error of Measurement (SEM) for all three devices. Clinically acceptable ICC values were set at no less than 0.707. The SEM for angle measurements should not exceed 5 degrees (8) and the SEM for strength should not exceed 2.69 kg, which is the Minimal Clinically Important Difference (MCID) for clinical use (9).

3. Usability of Software for Hand and Finger Movement Training:

Usability was assessed using the System Usability Scale (SUS) (10) with a total score out of 100 from 10 questions. SUS scores greater than 80.3 indicate excellent usability, scores between 68 and 80.3 indicate good usability, a score of 68 indicates average usability, scores between 51 and 68 indicate below-average usability, and scores below 51 indicate poor usability. (11)

### Confidentiality

The data records of all research participants will not include names, hospital numbers (HN), addresses, phone numbers, or any other identifiable information. Instead, a coding system will be used to maintain anonymity. Access to documents containing names and corresponding codes will be restricted to the research team members only. Electronic data will be password-protected to limit access. The data will be stored on computers in the Department of Rehabilitation Medicine, Faculty of Medicine, Chiang Mai University, for a period of 5 years before being permanently deleted. Additionally, the data will be backed up on the university's OneDrive, with access limited to the researchers via individual usernames and passwords.

### Quality assurance

Protocol and data protection process will be monitored by the EC committees.

### Expected outcomes of the study

Participants will not receive direct benefits from this research; however, the findings will contribute to the development of care systems and devices for individuals with arm weakness disabilities in the country.

Given the current situation, the provision of remote rehabilitation services for stroke patients has become essential, especially during the critical period of rehabilitation when recovery rates are highest. To maximize their ability to use their hands and care for themselves, patients need sufficient and effective hand rehabilitation from specialists, along with motivation to engage in training. Therefore, the research team believes that developing a remote hand function rehabilitation system using technology is crucial.

Such a system should be capable of physically assessing and monitoring patients, including measuring the range of motion and grip strength. It should offer systematically designed training programs and measure progress to tailor the therapy for everyone. Additionally, the system should effectively transmit useful data to rehabilitation professionals overseeing the patients' care.

### Dissemination of results and publication policy

The researcher plans to inform the study participants of the research results via email and phone. The findings will be published without revealing the identities of the participants. Subsequently, the researcher will begin planning tests with a group of stroke patients.

### Duration of the project

1 year

| **Activities** | **Month** | | | | | | | | | | | |
| --- | --- | --- | --- | --- | --- | --- | --- | --- | --- | --- | --- | --- |
|  | **1** | **2** | **3** | **4** | **5** | **6** | **7** | **8** | **9** | **10** | **11** | **12** |
| Manufacture of the prototypes and preparation of necessary documents |  |  |  |  |  |  |  |  |  |  |  |  |
| Ethical approval process |  |  |  |  |  |  |  |  |  |  |  |  |
| Data collection |  |  |  |  |  |  |  |  |  |  |  |  |
| Data analysis |  |  |  |  |  |  |  |  |  |  |  |  |
| Preparation of report |  |  |  |  |  |  |  |  |  |  |  |  |

### Problems anticipated

Ethical approval process could take longer than expected.

Available devices: since the prototypes are newly made. It is difficult to control the final design and the manufacturing time

### Project management

This section should describe the role and responsibility of each member of the team.

### Ethics

**Justification for the study, its significance**

Given the current situation, the provision of remote rehabilitation services for stroke patients has become essential, particularly during the critical period of recovery when the rate of rehabilitation is at its highest. To maximize their hand function and self-care abilities, patients need adequate and effective hand rehabilitation from specialists, along with motivation to engage in training. Therefore, the research team believes that developing a remote hand function rehabilitation system using technology is crucial.

Such a system should be capable of physically assessing and monitoring patients, including measuring range of motion and grip strength. It should offer systematically designed training programs and measure progress to tailor therapy for each individual. Additionally, the system should effectively transmit useful data to rehabilitation professionals overseeing the patients' care.

**Regulatory and Ethical Compliance**

“This research study is conducted in accordance with the ethical principles outlined in the Declaration of Helsinki (2013) and follows the guidelines presented in the CIOMS International Ethical Guidelines for Health-related Research Involving Humans (2016) and ICH GCP E6(R2).”

**Informed consent and participant recruitment**

A volunteer recruitment poster will be posted on the bulletin boards within the Department of Rehabilitation Medicine, Faculty of Medicine, Faculty of Science at Chiang Mai University, and on the Department of Rehabilitation Medicine's public website. Interested individuals can contact the researchers, Ms. Kanyaluck Uttarachon and/or Dr. Pornsuree Kuvijitsuwan, via email or phone to express their interest. They will then undergo an initial interview and screening assessment to determine eligibility according to the study criteria.

If the volunteers meet the criteria, the researchers will explain the project and answer any questions until the volunteers are satisfied before signing the informed consent form. Once signed, the volunteers will become research participants and will be assigned an identification number. Documents will list this identification number, and the names and personal information of the volunteers will remain confidential, accessible only to the relevant researchers. All electronic documents will be stored on password-protected computers at Chiang Mai University.

This research project selects healthy volunteers to test the prototype air guitar hand device before its actual use in stroke patients and other individuals with arm weakness.

### Ensuring the Welfare of Research Participants:

Research Injury Insurance Plan: The project does not provide research injury insurance or compensation for research-related injuries. However, if a participant is injured during the research, the researchers will provide close medical attention and care.

Facility, Equipment, and Personnel Preparedness: To ensure participant safety during the research, the testing of the prototype device will take place in the three-dimensional motion analysis room at the College of Arts, Media, and Technology, Chiang Mai University. The testing will be closely supervised by an occupational therapist.

### Informed consent forms

Informed consent forms in Thai and English are attached.

### References (of literature cited in preceding sections)

1. Langhorne P, Coupar F, Pollock A. Motor recovery after stroke: a systematic review. Lancet Neurol. 2009;8(8):741-54.

2. Koski L, Mernar T, Dobkin B. Immediate and Long-Term Changes in Corticomotor Output in Response to Rehabilitation: Correlation with Functional Improvements in Chronic Stroke. Neurorehabilitation and Neural Repair. 2004;18:230 - 49.

3. Langhammer B, Stanghelle JK. Bobath or motor relearning programme? A comparison of two different approaches of physiotherapy in stroke rehabilitation: a randomized controlled study. Clin Rehabil. 2000;14(4):361-9.

4. Samuelkamaleshkumar S, Reethajanetsureka S, Pauljebaraj P, Benshamir B, Padankatti SM, David JA. Mirror therapy enhances motor performance in the paretic upper limb after stroke: a pilot randomized controlled trial. Arch Phys Med Rehabil. 2014;95(11):2000-5.

5. Houwink A, Nijland RH, Geurts AC, Kwakkel G. Functional recovery of the paretic upper limb after stroke: who regains hand capacity? Arch Phys Med Rehabil. 2013;94(5):839-44.

6. Hughes A-M, Burridge JH, Demain SH, Ellis-Hill C, Meagher C, Tedesco-Triccas L, et al. Translation of evidence-based Assistive Technologies into stroke rehabilitation: users' perceptions of the barriers and opportunities. BMC Health Serv Res. 2014;14:124-.

7. Nittayasupaporn B, Tohsen F, Poungpum T, Apipunyasopon L. Development and assessment of CUFastTech mobile application for plain radiograph. The Thai Journal of Radiological Technology. 2021;45(1):1-7.

8. Jordan PW, Thomas B, McClelland IL, Weerdmeester B. Usability Evaluation In Industry: Taylor & Francis; 1996.

9. Norkin CC, White DJ. Measurement Of Joint Motion: A Guide To Goniometry: F.A. Davis Company; 2016.

10. Therapists ASoH, MacDermid J, Solomon G, Valdes K. Clinical Assessment Recommendations: American Society of Hand Therapists; 2015.

11. The NIHR Southampton Biomedical Research Centre (BRC). Procedure for Measuring Hand grip strength jusing the Jamar dynamometer. Southampton: NHS National Institute for Health Research 2016 May 2016.

12. Reissner L, Fischer G, List R, Taylor WR, Giovanoli P, Calcagni M. Minimal detectable difference of the finger and wrist range of motion: comparison of goniometry and 3D motion analysis. Journal of Orthopaedic Surgery and Research. 2019;14(1):173.

13. Fischer G, Jermann D, List R, Reissner L, Calcagni M. Development and Application of a Motion Analysis Protocol for the Kinematic Evaluation of Basic and Functional Hand and Finger Movements Using Motion Capture in a Clinical Setting—A Repeatability Study. Applied Sciences. 2020;10(18):6436.

Research protocol: part 2

Budget

| **Budget Categories** | **Details** | **Budget (Baht-THB)** |
| --- | --- | --- |
| Operating Budget:  Compensation | Travel compensation for volunteers [300 THB x 10 people]: 3,000 THB | 3,000 |
|  | Compensation for occupational therapists for demonstration assessments to be used in video games and applications [250 THB x 2 people x 10 sessions]: 5,000 THB | 5,000 |
| Salaries | Fee for composing therapeutic music, 10 songs: 20,000 THB | 20,000 |
|  | Fee for game production as designed, 1 project: 20,000 THB | 20,000 |
|  | Fee for developing an application for Android, iOS, and website, 1 project: 80,000 THB | 80,000 |
|  | Fee for designing prototype device Version 2 for improved usability, from prototypes Version 1.1 and 1.2: 25,000 THB | 25,000 |
|  | Fee for producing 5 prototype devices: 30,000 THB | 30,000 |
| Materials | Office supplies: 2,000 THB | 2,000 |
| Operational Expenses | Cloud server rental: 10,000 THB | 10,000 |
|  | apple developer account fee 5000THB | 5,000 |
| Capital Budget  Equipment | None |  |
| Total |  | 200,000 |

Other support for the project

This work is funded by the Faculty of Medicine, Chiang Mai University (Grant number 356/2022) and National Research Council of Thailand (NRCT).

Collaboration with other scientists or research institutions

Links to other projects

Curriculum Vitae of investigators

CVs of the investigators are attached.

Other research activities of the investigators

None

Financing and insurance

This work is funded by the Faculty of Medicine, Chiang Mai University (Grant number 356/2022) and National Research Council of Thailand (NRCT). None of insurance company/organization is involved.

The project does not provide research injury insurance or compensation for research-related injuries. However, if a participant is injured during the research, the researchers will provide close medical attention and care.
